# Clinical Features of Clostridial Necrotizing Soft-tissue Infections: Analysis of a Prospective Scandinavian Cohort Study

**DOI:** 10.1093/ofid/ofag429

**Published:** 2026-07-16

**Authors:** Jesper Andreas Viste, Gard Andreas Svendal, Steinar Skrede, Ole Hyldegaard, Michael Nekludov, Per Arnell, Ylva Karlsson, Anna Norrby-Teglund, Trond Bruun

**Affiliations:** Department of Clinical Science, University of Bergen, Bergen, Norway; Department of Clinical Science, University of Bergen, Bergen, Norway; Department of Clinical Science, University of Bergen, Bergen, Norway; Department of Medicine, Haukeland University Hospital, Bergen, Norway; Department of Anaesthesiology, Centre of Head and Orthopaedics, Hyperbaric Unit, University Hospital of Copenhagen, Rigshospitalet, Copenhagen, Denmark; Department of Clinical Medicine, University of Copenhagen, Copenhagen, Denmark; Perioperative Medicine and Intensive Care, Intensive Care and Thoracic Surgery, Hyperbaric Medicine, Karolinska University Hospital Solna, Stockholm, Sweden; Department of Anesthesia and Intensive Care Medicine, Sahlgrenska University Hospital, Gothenburg, Sweden; Department of Anaesthesia and Intensive Care, Blekinge County Hospital, Karlskrona, Sweden; Center for Infectious Medicine, Karolinska Institutet, Karolinska University Hospital Huddinge, Stockholm, Sweden; Department of Clinical Science, University of Bergen, Bergen, Norway; Department of Medicine, Haukeland University Hospital, Bergen, Norway

**Keywords:** gas gangrene, Clostridial myonecrosis, necrotizing soft tissue infection, Clostridium perfringens, Clostridium septicum

## Abstract

**Background:**

Necrotizing soft-tissue infections (NSTI) caused by *Clostridium* spp. are rare. High rates of amputation and mortality are reported, but knowledge on clinical characteristics and outcome is largely based on retrospective cohorts. The role of *Clostridium* spp. as part of polymicrobial NSTI is also unclear.

**Method:**

We identified all clostridial cases from the INFECT cohort, a prospective study including patients with confirmed NSTI admitted to 5 Scandinavian referral hospitals over a 4-year period. Selected parameters were compared to a control group of nonclostridial polymicrobial infections. Additionally, we analyzed polymicrobial infections with clostridial species.

**Results:**

Ten cases of clostridial NSTIs were identified, evenly divided between cases caused by *Clostridium perfringens* and *Clostridium septicum*. Immunodeficiency (4/10) and penetrating trauma (3/10) were significantly more frequent compared to polymicrobial controls. Clostridial NSTI was also associated with extremity involvement (10/10) and severe pain (7/9). Radiological evidence of gas (4/7) did not distinguish clostridial from polymicrobial infections. Two patients underwent amputation, and 2 others died within 30 days. The cohort also included seven polymicrobial NSTIs with *Clostridium* spp., with higher rates of both comorbidity (7/7) and 30-day mortality (4/7).

**Conclusions:**

Clostridial NSTIs were associated with extremity infection, immunodeficiency, and penetrating trauma. The cases demonstrate a wide spectrum of severity. In polymicrobial NSTIs involving *Clostridium* spp., the mortality was particularly high, but the pathogenetic role of clostridia in these infections is unclear.

## Teaser

In a large prospective cohort of patients with necrotizing soft tissue infections, clostridial infections were associated with extremity involvement, immunodeficiency, and penetrating trauma. Amputation and mortality rates were lower than those typically reported, reflecting a wide spectrum of severity.

Clostridial gas gangrene is a rare but devastating type of necrotizing soft-tissue infection (NSTI). Rapid progression and high mortality and amputation rates have been reported [1–4]. Primary muscle involvement is typical, hence the term clostridial myonecrosis. Other types of NSTI, most frequently streptococcal or mixed aerobic/anaerobic infections, are primarily affecting skin and subcutaneous tissue and are often classified as necrotizing fasciitis.

The incidence of NSTI is uncertain and highly variable across studies and geographical regions. In Europe, recent studies report an incidence approximately 1–2 cases per 100 000 [5–7], and some studies suggest that it is increasing [5, 6]. A small fraction of NSTIs is caused by bacteria from the *Clostridium* genus. Clostridia are gram-positive anaerobic rods, and species associated with NSTI include *Clostridium perfringens*, *Clostridium septicum, Clostridium histolyticum, Clostridium novyi*, and *Clostridium sordellii* [2]. As for *Streptococcus pyogenes*, a more frequent cause of NSTI, clostridia have an innate ability to rapidly secrete a wide range of toxins playing a major role in the pathogenesis [8, 9].

Previous data on clostridial NSTI consists of case reports and case series, which suggest that an aggressive course and high mortality is typical of clostridial myonecrosis. Rapid and resource demanding treatment is required, and early amputation may be lifesaving [7, 10]. Swift recognition, and timely and tailored treatment, could possibly be improved by better knowledge of early clinical characteristics indicating clostridial etiology.

There are few established clinical features that may discriminate clostridial cases in the early stages from other NSTI, although clostridial NSTI have been associated with severe systemic toxicity, lower extremity infection, and gas in tissue [2, 11]. Predisposing factors may also give clues to the etiology. Contaminated wounds after traumatic injury or gastrointestinal disease are common in clostridial infection but may also be seen in polymicrobial NSTI.

In this study, we use data from a large prospective Scandinavian cohort of NSTI to validate previous findings, hypothesizing that certain risk factors and clinical features differ between clostridial and similar NSTI cases. Our cohort made it possible to directly and statistically compare clostridial and a control group of polymicrobial NSTI. The prospective data also give an overview of an unselected and possibly representative group of clostridial NSTI, not only classical myonecrosis, supplementing retrospective case studies where less severe cases probably often can be omitted. Finally, we wanted to describe the characteristics of cases were clostridia are identified as a part of a polymicrobial infection.

## METHODS

### Setting and Patients

This work is a substudy based on data from the INFECT project (clinicalTrials.gov (NCT01790698)), a multicenter, observational cohort study of Scandinavian patients with NSTIs, in which a total of 409 cases were included. Details about the study design, general patient inclusion criteria, study hospitals, and study ethics have been described previously [11]. In short, adult patients (≥18 years) with suspected NSTI were prospectively enrolled in 5 Scandinavian referral hospitals providing hyperbaric oxygen therapy (HBOT) between February 2013 and June 2017. The presence of NSTI was determined by the surgeon and based on findings of necrotic or deliquescent soft tissue with undermining of the surrounding tissue [11]. Patients were treated according to local protocols. In this substudy, clostridial cases, defined by identification of a clostridium species as the sole pathogen, were selected for further analysis. In addition, selected analyses were performed in cases where clostridia were identified as part of a polymicrobial etiology.

For comparison with the clostridial cases, we chose a control group consisting of nonclostridial polymicrobial infections from the same cohort of NSTI patients because these are most frequent and commonly contain other anaerobic microbes. We excluded polymicrobial cases involving *S. pyogenes* as we deemed it likely that these were, in effect, streptococcal infections, with their unique pathogenesis and clinical features, described elsewhere [12].

Furthermore, we categorized the clostridial cases into 2 subgroups: spontaneous or traumatic/postoperative. A patient was categorized as traumatic/postoperative if there was a history of penetrating trauma, blunt trauma, or surgery during the 4 weeks prior to the NSTI. All other cases were categorized as spontaneous.

### Clinical Parameters

Selected variables from the INFECT study were chosen to describe possible risk factors, clinical features, disease severity, treatment, and outcome. The parameters were chosen based on traits known from literature and the author's clinical experience with these infections. The collection of clinical and laboratory parameters is previously described in detail [11]. All data in the INFECT study were registered prospectively. The most abnormal preoperative laboratory test values were used. As indicators of disease severity, presence of septic shock, simplified acute physiology score (SAPS) II and sequential organ failure assessment (SOFA) score, were registered. The SOFA scoring was modified because cerebral failure was not assessed. The laboratory risk indicator for necrotizing fasciitis (LRINEC) score was also calculated based on preoperative measurements [13].

### Microbiology

Microbiological testing was done according to local laboratory protocols. Findings were categorized according to the distinct species identified by culture of blood or tissue samples [14]. These were taken from normally sterile sites during surgery. The microbiological category *Clostridium* spp. included cases where the laboratory identified clostridial species but did not provide further specification or where subspecies other than *C. perfringens* or *C. septicum* were identified.

### Statistical Analyses

Data were analyzed using IBM SPSS Statistics, version 30. For statistical comparison, we selected a small number of parameters based on key aspects of the patient course to ensure clinical relevance and limit multiple comparison issues. We restricted this testing to compare monomicrobial clostridial NSTI and the control group. Categorical variables were analyzed using Fisher's exact test and continuous variables were compared using the Mann-Whitney *U* test. A 2-sided *P* < .05 was considered statistically significant.

## RESULTS

### Microbiological Etiology

A total of 10 clostridial NSTI cases were identified among the 409 cases in the INFECT cohort, evenly divided between cases with *C. perfringens* and *C. septicum* (Supplementary table 1). Six of the cases had positive blood cultures. In addition, clostridia were identified in 7 polymicrobial infections, 1 with *C. perfringens* grown from blood.

### Demographics and Risk Factors

Overall, patient demographics were similar in clostridial NSTI and the nonclostridial polymicrobial controls, with a mean age of 60 years (Table 1). Comorbidity was recorded in 8 of 10 clostridial cases, most commonly cancer (5/10; 2 cases with *C. perfringens* and 3 cases with *C. septicum*) or any cause of immunodeficiency (4/10; 3 cases with *C. perfringens* and 1 case with *C. septicum*). Few patients had other severe chronic diseases. Compared to the control group, significantly higher proportions of the patients were immunodeficient, had cancer, or had a preceding penetrating trauma (Table 2). Risk factors and comorbidities such as high alcohol consumption, diabetes mellitus, and cardiovascular or peripheral vascular disease, were observed more frequently in the control group (Table 1). Four cases occurred after trauma or surgery, whereas 6 cases were defined as spontaneous (Table 3).

**Table 1.** Demographics, Comorbidities and Other Risk Factors in Clostridial NSTI, Polymicrobial NSTI With Clostridia, and Nonclostridial NSTI Controls

	Monomicrobial (N = 10)^a^	Polymicrobial (N = 7)^b^	Controls (N = 185)^c^
Demographics			
Age, mean	62 (21–87)	56 (21–72)	59 (23–88)
Sex, male	5 (50)	6 (86)	113 (61)
BMI^d^, kg/m^2^	27 (15–37)	25 (20–29)	27 (15–56)
Active smoking^e^	3 (38)	2 (33)	73 (47)
Excessive alcohol use^f,g^	0 (0)	2 (40)	28 (20)
Previous NSTI	0 (0)	1 (14)	1 (.5)
Comorbidities			
Immunodeficiency^h^	4 (40)	2 (29)	19 (10)
Diabetes mellitus, type 1 and 2	0 (0)	3 (43)	69 (37)
Active malignancy^i^	3 (30)	0 (0)	15 (8)
Hematological malignancy	2 (20)	1 (14)	3 (2)
Chronic kidney disease	1 (10)	2 (29)	20 (11)
Chronic obstructive pulmonary disease	0 (0)	1 (14)	25 (14)
Cardiovascular disease^j^	1 (10)	3 (43)	83 (45)
Peripheral vascular disease	1 (10)	3 (43)	27 (15)
Chronic liver disease	0 (0)	0 (0)	10 (5)
Rheumatoid disease	0 (0)	1 (14)	6 (3)
No comorbidity^k^	2 (20)	0 (0)	45 (24)
Preceding events or skin breaches			
Surgery^l^	2 (20)	2 (29)	51 (28)
Penetrating trauma^l^	3 (30)	0 (0)	7 (4)
Blunt trauma^l^	0 (0)	1 (14)	13 (7)
Intravenous drug use	1 (10)	0 (0)	12 (6)
Varicella zoster	0 (0)	0 (0)	1 (.5)
Chronic wound or other skin disease	1 (10)	1 (14)	19 (10)
None of the factors listed above	4 (40)	4 (57)	100 (54)

Unless otherwise specified, data are presented as median (total range) or number (%).

Abbreviations: BMI, body mass index; NSTI, necrotizing soft-tissue infection.

^a^Clostridial NSTI.

^b^Polymicrobial NSTI with clostridia.

^c^Selected group of nonclostridial polymicrobial NSTIs from the INFECT cohort.

^d^Data missing for 4 patients in the control group.

^e^Data missing for 2 patients in the monomicrobial group, 1 patient in the polymicrobial group, and 29 patients in the control group.

^f^Excessive alcohol use was defined as weekly intake of >14 units for women and >21 for men.

^g^Data missing for 3 patients in the monomicrobial group, 2 patients in the polymicrobial group, and 43 patients in the control group.

^h^Includes any immunodeficiency or use of steroids/other immunosuppressive drugs.

^i^Active malignancy was defined as malignancy other than hematological, and that had not been eradicated.

^j^Includes, but not limited to, hypertension, myocardial infarction, angina pectoris, heart failure, apoplexia.

^k^None of the conditions above.

^l^Within the past 4 weeks before NSTI.

**Table 2.** Comparison of Clostridial NSTI and Nonclostridial Polymicrobial Controls

Clinical Parameters	Clostridial NSTI (N = 10)^a^	Controls (N = 185)^b^	*P* Value
Patient demographics and risk factors			
Age, mean	62 (21–87)	59 (23–88)	.350
Sex, male	5 (50)	113 (61)	.520
Cancer^c^	5 (50)	16 (9)	.**002**
Immunodeficiency^d^	4 (40)	19 (10)	.**019**
No comorbidity^e^	2 (20)	45 (24)	1.000
Penetrating trauma	3 (30)	7 (4)	.**010**
Clinical features			
Primary site of infection			**<.001**
Trunk + head/neck	0 (0)	153 (83)	
Extremities	10 (100)	32 (17)	
Severe pain^f^	7 (78)	75 (42)	.**044**
Lactate (mmol/L)^g^	1.9 (0.7–7.6)	1.5 (0.5–19.0)	.728
Hemoglobin (g/dL)^h^	9.4 (7.0–13.4)	11.4 (5.8–21.1)	.067
Septic shock	6 (60)	81 (44)	.346
LRINEC^i^	7 (1–10)	8 (0–13)	.582
Gas on radiology^j^	4 (57)	84 (60)	1.000
Treatment and outcome			
Mechanical ventilation^k^	9 (90)	173 (94)	.507
Hyperbaric oxygen therapy^l^	7 (70)	161 (87)	.146
Amputation^m^	2 (22)	9 (17)	1.000
30-day mortality^n^	2 (20)	22 (12)	.357

Unless otherwise specified, data are presented as median (total range) or number (%). Boldface indicates statistical significance.

Abbreviations: LRINEC, laboratory risk indicator for necrotizing fasciitis; NSTI, necrotizing soft-tissue infection.

^a^Clostridial NSTI.

^b^Selected group of nonclostridial polymicrobial NSTIs from the INFECT cohort.

^c^Either active malignancy (defined in Table 1) or hematological malignancy.

^d^Includes any immunodeficiency or use of steroids/other immunosuppressive drugs.

^e^None of the following: active malignancy, chronic obstructive pulmonary disease or asthma, current or previous cardiovascular disease, peripheral vascular disease, diabetes mellitus, chronic kidney failure, chronic liver disease, rheumatoid disease, immunodeficiency/immunosuppression.

^f^Data missing in 1 patient in the monomicrobial group and in 7 patients in the control group.

^g^Highest preoperative values observed. Data missing for 2 patients in the monomicrobial group and 50 patients in the control group.

^h^Lowest preoperative values observed. Data missing for 9 patients in the control group.

^i^Data missing for 24 patients in the control group.

^j^Data missing for 3 patients in the monomicrobial group and 46 patients in the control group.

^k^During first 7 d of stay at intensive care unit or high-dependency unit.

^l^At any time during hospitalization.

^m^Amputation of limb among cases with extremity affection at primary hospital or during first 7 d of stay at intensive care unit or high-dependency unit at study hospital. Data missing for 1 patient in the monomicrobial group.

^n^Data missing for 1 patient in the control group.

**Table 3.** Spontaneous versus Traumatic/Postoperative Clostridial NSTI

	Spontaneous NSTI (N = 6)	Traumatic/Postoperative NSTI (N = 4)
Microbiological agent		
*C. perfringens* cases^a^	2 (33)	3 (75)
*C. septicum* cases^a^	4 (67)	1 (25)
Demographics and risk factors		
Age, mean	68 (51–87)	61 (21–77)
Sex, male	3 (50)	2 (50)
BMI	26 (15–30)	31 (24–37)
Previous NSTI	0 (0)	0 (0)
Excessive alcohol use^b^	0 (0)	0 (0)
Active smoking^c^	1 (17)	2 (50)
Comorbidities		
Immunocompromised^d^	3 (50)	1 (25)
Diabetes mellitus, type 1 and 2	0 (0)	0 (0)
Active malignancy	2 (33)	1 (25)
Hematological malignancy	2 (33)	0 (0)
Chronic kidney disease	0 (0)	1 (25)
Chronic obstructive pulmonary disease	0 (0)	0 (0)
Cardiovascular disease	1 (17)	0 (0)
Peripheral vascular disease	0 (0)	1 (25)
Rheumatoid disease	0 (0)	0 (0)
No comorbidity^e^	1 (17)	1 (25)
Clinical features and primary site of infection		
Septic shock	3 (50)	3 (75)
SOFA score day 1^f^	8 (1–14)	7 (3–14)
Head/neck	0 (0)	0 (0)
Upper extremity	1 (17)	1 (25)
Abdomen, anogenital	0 (0)	0 (0)
Lower extremity	5 (83)	3 (75)
Outcome		
Amputation^g^	1 (17)	1 (25)
30-day mortality	2 (33)	0 (0)
90-day mortality	3 (50)	1 (25)

Unless otherwise specified, data are presented as median (total range) or number (%).

Abbreviations: BMI, body mass index; NSTI, necrotizing soft-tissue infection.

^a^Confirmed by culture of either blood or tissue sample.

^b^Excessive alcohol use was defined as weekly intake of >14 units for women and >21 for men. Data missing in 3 spontaneous cases.

^c^Data missing in 3 spontaneous cases.

^d^Includes any immunodeficiency or use of steroids/other immunosuppressive drugs.

^e^None of the conditions above.

^f^SOFA score includes subscores ranging from 0 to 4 for each of 5 components (circulation, lungs, liver, kidneys, and coagulation). Aggregated scores range from 0 to 20, with higher scores indicating more severe organ failure. The scoring was modified because cerebral failure was not assessed. Data missing in 1 spontaneous case.

^g^Amputation of limb at primary hospital or during first 7 d of stay at intensive care unit or high-dependency unit at study hospital.

### Clinical Features

All clostridial infections were located in the extremities, with 2 affecting the upper and 8 the lower extremities (Supplementary table 2). No cases involved the head or neck region. Preoperative findings of skin bullae, bruising, and purple discoloration were seen in approximately one third. Severe pain was recorded in 7 of 9 evaluable cases. Six patients had septic shock.

Compared to the control group, clostridial infections were associated with extremity infection (*P* < .001) and they had a higher prevalence of severe pain (*P* = .044) (Table 2). Although not statistically significant, they also had a higher rate of septic shock (60% vs 43%) and a lower median hemoglobin value (9.4 vs 11.4). Preoperative levels of C-reactive protein (CRP) and leukocytes were lower than in the controls, whereas lactate levels and SAPS and LRINEC scores were slightly more elevated (Supplementary Table 2). SOFA scores were similar. Findings on radiological imaging suggestive of gas formation were reported at comparable rates around 60%.

### Treatment and Outcome

There were no clear differences observed in the treatment modalities comparing clostridial cases and the control group (Table 2 and Supplementary table 3). Nine clostridial cases required mechanical ventilation and 2 needed renal replacement therapy, none of which had preexisting kidney disease. Seven of the cases received HBOT. However, 2 of the patients who did not receive this treatment option were dead within 48 hours after diagnosis. Among the patients with affection of the extremities, 22% (2/9) required amputation, compared to 16% (9/54) in the control group (*P* = 1.00). The 30-day mortality rate was 20% versus 12% (*P* = .357). The mortality rate rose to 40% after 90 days in clostridial cases and 18% in controls.

### Polymicrobial NSTI With Clostridium

Among 7 patients with polymicrobial infections where *Clostridium* spp. were identified, none were without comorbidities, as opposed to nearly one fourth of the control group (Table 1). Diabetes mellitus and cardiovascular disease was particularly common in both these polymicrobial groups. The polymicrobial cases with clostridia presented with typical skin findings more frequently than monomicrobial clostridial infections, along with more pronounced elevation in CRP and leukocytes (Supplementary Table 2). The infections were most often located in the abdominal or anogenital region (4/7), but lower extremity infection was also common (3/7). Overall, this group had the highest observed 30-day mortality rate (4/7).

## DISCUSSION

The primary aim of this study was to describe characteristics of clostridial NSTIs and identify clinical features indicative of clostridial etiology. The clostridial cases comprised 10 of the 409 NSTI cases included in the INFECT cohort, confirming the rarity of these infections. As hypothesized, several risk factors and clinical features were associated with clostridial etiology, including immunodeficiency, penetrating trauma, and extremity infection. Severity was high, as in other NSTIs, and the SAPS II score was slightly higher compared to the nonclostridial controls. Even so, our data indicate that clostridial cases may present with a broader clinical spectrum, often less severe than typical descriptions of gas gangrene.

The higher rate of *C. septicum* compared to previous reports might be explained by the number of spontaneous cases, of which most infections were caused by *C. septicum*. Along with improved sanitary conditions and less manual labor, the rising incidence of colon cancer, may have caused a shift from *C. perfringens* toward *C. septicum*.

Clostridia require an entry point, a premise that might underlie the risk factors associated with clostridial NSTI. Infections caused by *C. perfringens* have historically been associated with deep penetrating trauma, whereas *C. septicum* has been linked to spontaneous cases afflicting patients with gastrointestinal malignancy or immunodeficiency [1, 2, 15, 16]. In a recent case series of 5 cases by Leiblein et al, none had penetrating trauma, and the cases were either spontaneous or related to decubitus or surgery [4]. This probably reflects changes in epidemiology, as less cases are related to war and suboptimal trauma management than what has been seen historically. In our study, however, penetrating trauma was seen in several of the cases with *C. perfringens*, and significantly more often than in the controls, suggesting that possible clostridial etiology should still be suspected in NSTI following penetrating injuries. Such trauma pose a particular risk for clostridial infections due to contamination of wounds as well as the risk of compromised blood supply, creating ideal conditions for spore-forming anaerobic bacteria from the environment [1]. With respect to other risk factors like underlying malignancy or immunodeficiency, we found no consistent association with specific clostridium species, but the majority of patients with these risk factors had spontaneous infections, without preceding trauma.

None of the patients with clostridial infections had diabetes mellitus, despite it being reported as a common comorbidity in spontaneous clostridial NSTIs [4, 15].

Unexpectedly, a low frequency of clostridial NSTIs presented with skin findings described as typical in the literature [2], different to polymicrobial cases with clostridia [2]. Cases lacking such skin findings were also seen in the previously mentioned recent study, however [4]. A possible explanation may be that clostridial infections develop fast, with patients often hospitalized before skin findings become evident. This is supported by similar results among cases caused by *S. pyogenes*, another rapidly progressing type of NSTI [12]. Additionally, retrospective case reports might be skewed toward patients presenting with classical findings of gas gangrene.

Severe pain is often described as the initial symptom, especially in spontaneous cases [15]. Strong pain was also seen in all 5 patients in the case series by Leiblein et al [4]. Among our patients, severe pain was significantly more frequent in clostridial cases compared to the control group. In a previous INFECT article describing *S. pyogenes* NSTIs, a circumstance resembling clostridial infections, severe pain was seen in only 45% of cases [12]. We speculate that the higher prevalence of pain in clostridial NSTIs might be linked to their specific pathogenesis, where secretion of alpha toxin induces formation of occluding microthrombi, subsequently causing ischemia [8].

Microbiological etiology in NSTI is strongly related to site of infection [17, 18]. As reported previously from the INFECT study, there was a clear association between clostridial NSTI and infection located to the extremities [11].

The detection of gas within the afflicted tissue, either by palpation or radiological imaging, has been viewed as a classical finding in clostridial NSTI cases. However, as gas may be produced by various unrelated microbes, the presence of gas is also common in polymicrobial infections [1, 19]. Both in the INFECT cohort and a recent study of foot infections, polymicrobial etiology was much more frequent than *Clostridium* spp. as cause of gas producing soft-tissue infections [11, 19]. We found that palpable crepitus occurred more often in clostridial infections than in the control group (44% vs 23%), whereas radiological imaging showed gas in similar proportions for both groups (57% vs 60%). These results point to gas in tissue as a nonspecific sign regarding the differentiation of clostridial and polymicrobial NSTI, with low sensitivity in clinical identification of clostridial NSTIs.

In clostridial cases, 60% of patients developed shock, comparable to the rate seen in NSTIs caused by *S. pyogenes* [12]. Circulatory failure has also been described in other studies and accords with the systemic effects of clostridial toxins [4, 8, 9]. Although leukocytosis was common in our clostridial cases, the values were lower than in the controls. This opposes previous findings associating clostridial NSTIs with pronounced leukocytosis at hospitalization [3, 4]. Also contrary to earlier findings, the clostridial cases had lower CRP levels [4]. Interestingly, the hemoglobin levels were lower in the clostridial cases, which might represent a diagnostic clue and may be due to the hemolytic effects of alpha toxin [20]. The high rates of malignancy may be a confounder, as median hemoglobin (g/dL) in clostridial NSTIs with or without cancer was 9.0 (7.0–10.7) versus 9.7 (8.4–13.4), respectively.

In 2005, Anaya et al found that *Clostridium* was a predictor of increased risk of limb loss with an odds ratio of 3.9 [3]. We found no significant difference between the groups in amputation rates. The number of surgeries were not higher either, supporting that these infections may not always be as severe as previously thought.

All study hospitals provided HBOT. There is uncertainty regarding its role in the treatment of NSTIs, reflected in the literature with retrospective data showing mixed results [21–24]. Given the obligate anaerobic nature of *Clostridium* spp., HBOT is by several authors seen as a promising or even mandatory adjunctive therapy, especially for this type of NSTI. A randomized controlled trial on HBOT in NSTI is needed, and recruiting started in February 2026 in such a study (clinicalTrials.gov; NCT07489274). Notwithstanding, given the low frequency, it will be difficult to include enough clostridial cases to evaluate the efficiency specifically for this group.

The mortality rate of NSTI overall ranges between 10% and 30%, and depends on multiple parameters, including age, comorbidities, and microbial etiology [6, 7, 17, 25]. The wide array of microbes causing NSTIs, combined with the rarity of the condition, makes it difficult to estimate the differences in mortality rate attributable to specific etiology. A recent metaanalysis of prognostic factors did not have sufficient data on etiology to identify associations to outcome [26]. Lower rates of mortality have been reported in *S. pyogenes* NSTI, probably because of younger age and fewer comorbidities in this group [11]. Traumatic clostridial NSTI has an estimated mortality rate close to that of NSTIs as a whole, whereas mortality as high as 70% has been reported for spontaneous clostridial myonecrosis [15, 27]. This is probably strongly related to underlying cancer or immunodeficiency. In our study, 30-day mortality of clostridial NSTI was lower than what is reported in the literature [1, 3, 4], possibly related to prospective data collection leading to a broader spectrum of disease severity in our cohort.

In polymicrobial NSTI in general, the pathogenesis is probably largely driven by microbial interplay and proteolytic activity, and less dependent on specific toxin effects like in streptococcal and clostridial infections [1, 28]. Notably, the combination of microbes is likely not random but rather shaped by synergistic interactions between specific species [28]. The burden of comorbidity among the patients with polymicrobial infections involving clostridia was high, in line with what we already know of type 1 NSTIs [1]. These 7 cases had not only comorbidities, but also evidence of high-severity NSTI, as all were mechanically ventilated and received HBOT, 5 of 7 had vasopressor treatment and 6 of 7 had 3 or more surgeries (data not shown). Comorbidity may be the main factor behind the high case-fatality rate, but it cannot be excluded that virulence of *Clostridium* spp. also contributes to disease severity.

The strength of this study lies in the design of the INFECT study, with a standardized protocol for data collection and unselected, prospective enrollment at multiple centers. Although the INFECT study is the largest of its kind to date, the primary limitation of our study is related to the relatively small number of clostridial cases, which restricts the power and feasibility of statistical analysis. However, to the best of our knowledge, there are no other studies on the subject with prospective patient enrollment, and previous data comprised case reports and small retrospective studies. Because of the limited sample size, we reduced the number of statistical analyses and focused primarily on describing the observed data. That all study sites were regional hospitals for treatment of NSTI might introduce a selection bias, where patients too unstable to transfer or patients with less severe infections were not included. Other limitations are related to variations among the laboratories with respect to how extensive species identification was performed in polymicrobial infections. This has impacted the stringency of the polymicrobial groups, and also the number of cases available for comparing different clostridial species. In addition, some variables that are of particular interest in clostridial infection were unavailable, such as types of malignancy and hemolysis parameters.

In summary, data from the prospective INFECT study support immunodeficiency and penetrating trauma as predisposing risk factors for clostridial NSTI. Infection of extremities was typical. Most patients presented with severe pain, but frequently without classical skin findings. Gas on radiology could not differentiate between clostridial and polymicrobial infections. The amputation and mortality rates were lower than in most previous studies, possibly because of prospective inclusion and a wider spectrum of cases. In polymicrobial NSTIs with clostridia involved, mortality was particularly high, but so was the number of comorbidities.

## Supplementary Material

ofag429_Supplementary_Data

## Supplementary Data

Supplementary materials are available at *Open Forum Infectious Diseases* online. Consisting of data provided by the authors to benefit the reader, the posted materials are not copyedited and are the sole responsibility of the authors, so questions or comments should be addressed to the corresponding author.
