# Supplementary material for: Clinical Features of Clostridial Necrotizing Soft-tissue Infections: Analysis of a Prospective Scandinavian Cohort Study
Source: Open Forum Infect Dis. 2026 Jul 16;13(8):ofag429. doi: 10.1093/ofid/ofag429 (PMC13435621; doi:10.1093/ofid/ofag429)
Supplement: Supplementary file 1 [file ofag429_supplementary_data.docx]

**Supplementary material for:**

**Clinical Features of Clostridial Necrotizing Soft-Tissue Infections: Analysis of a Prospective Scandinavian Cohort Study**

Jesper Andreas Viste, Gard Andreas Svendal, Steinar Skrede, Ole Hyldegaard, Michael Nekludov, Per Arnell, Ylva Karlsson, Anna Norrby-Teglund, and Trond Bruun

**Contents**

Supplementary Table 1. Microbiology	1

Supplementary Table 2. Clinical Features	2

Supplementary Table 3. Treatment Outcome	3

| **Supplementary Table 1. Microbiology** | | |
| --- | --- | --- |
|  | **Monomicrobial (N = 10)^a^** | **Polymicrobial (N = 7)^b^** |
| *Microbiological agent* |  |  |
| *C. perfringens* | 5 (50) | 1 (14) |
| *C. septicum* | 5 (50) | 0 (0) |
| *Clostridium* spp*.*^c^ | 0 (0) | 6 (86) |
| *Sample source* |  |  |
| Blood culture | 6 (60) | 4 (40)^d^ |
| Tissue sample | 9 (90) | 7 (100) |

Data are presented as number (%). Abbreviations: NSTI, Necrotizing soft-tissue infection. C., *Clostridium*.

^a^Clostridial NSTI.

^b^Polymicrobial NSTI with clostridia.

^c^Laboratory confirmed Clostridium not identified at species level or other Clostridial spp. than *C. perfringens* or *C. septicum*.

^d^*C. perfringens* was specified in 1 case. Other clostridia than *C. perfringens* and *C. septicum* were detected in 2 cases.

In 1 case was not specified to species level. In 2 of the cases, other agents were also detected in blood culture together with

clostridia: 1 case with coagulase-negative staphylococci and 1 case with *Bacteroides* spp.

| **Supplementary Table 2. Clinical Features** | | | |
| --- | --- | --- | --- |
|  | **Monomicrobial (N = 10)^a^** | **Polymicrobial (N = 7)^b^** | **Controls (N = 185)^c^** |
| *Primary site of infection* |  |  |  |
| Head/Neck | 0 (0) | 0 (0) | 42 (23) |
| Upper extremity | 2 (20) | 0 (0) | 3 (2) |
| Abdomen, anogenital | 0 (0) | 4 (57) | 111 (60) |
| Lower extremity | 8 (80) | 3 (43) | 29 (16) |
| *Severity* |  |  |  |
| Septic shock^d^ | 6 (60) | 3 (43) | 81 (44) |
| SOFA score day 1^e^ | 7 (1-14) | 10 (4-16) | 7 (0-16) |
| SAPS II^f^ | 47 (22-82) | 48 (29-54) | 41 (15-104) |
| LRINEC score (0-13)^g^ | 7 (1-10) | 9 (4-13) | 8 (0-13) |
| Muscle affected^h^ | 7 (70) | 4 (57) | 117 (63) |
| *Preoperative symptoms and signs* |  |  |  |
| Severe pain^i^ | 7 (78) | 5 (83) | 75 (42) |
| Skin bullae^j^ | 3 (33) | 5 (71) | 42 (23) |
| Purple/Black skin discoloration^k^ | 3 (30) | 4 (57) | 55 (32) |
| Skin bruising^l^ | 3 (33) | 5 (83) | 89 (51) |
| Skin anesthesia^m^ | 1 (14) | 1 (25) | 10 (8) |
| Palpable crepitus^n^ | 4 (44) | 4 (100) | 36 (23) |
| *Laboratory and radiological findings^o^* |  |  |  |
| Leukocytes (x 10^9^/L)^p^ | 13.4 (.2-49.0) | 15.3 (.1-27.5) | 17.7 (.3-56.4) |
| CRP (mg/L)^q^ | 169 (42-300) | 450 (98-657) | 310 (9-700) |
| Lactate (mmol/L)^r^ | 1.9 (0.7-7.6) | 2.4 (1.2-4.9) | 1.5 (0.5-19.0) |
| Creatinine (μmol/L)^s^ | 87 (39-217) | 168 (48-332) | 103 (18-958) |
| Hemoglobin (g/dL)^t^ | 9.4 (7.0-13.4) | 9.1 (5.1-15.0) | 11.4 (5.8-21.1) |
| Gas on radiology^u^ | 4 (57) | 3 (75) | 84 (60) |

Data are presented as median (total range) or number (%). Abbreviations: LRINEC, laboratory risk indicator for necrotizing fasciitis; NSTI, necrotizing sof-tissue infection; SAPS, simplified acute physiology score; SOFA, sequential organ failure assessment.

^a^Clostridial NSTI.

^b^Polymicrobial NSTI with clostridia.

^c^Selected group of non-clostridial polymicrobial NSTIs.

^d^Septic shock was defined as s-lactate > 2 mmol/l and use of vasopressor or inotrope.

^e^SOFA score includes subscores ranging from 0 to 4 for each of 5 components (circulation, lungs, liver, kidneys, and coagulation). Aggregated scores range from 0 to 20, with higher scores indicating more severe organ failure. The scoring was modified because cerebral failure was not assessed. Data missing for 1 patient in the polymicrobial group and 10 patients in the control group.

^f^SAPS II is calculated the first 24 hours in the intensive care unit/high-dependency unit from 17 variables; scores range from 0 to 163, with higher scores indicating more severe disease. Data missing for 1 patient in the monomicrobial group, 2 patients in the polymicrobial group and 16 patients in the control group.

^g^Data missing for 1 patient in the polymicrobial group and 24 patients in the control group.

^h^Assessed during surgery in the primary hospital or during first 7 days of stay at intensive care unit or high-dependency unit at specialized hospital.

^i^Data missing for 1 patient in the monomicrobial group, 1 patient in the polymicrobial group and 7 patients in the control group.

^j^Data missing for 1 patient in the monomicrobial group and 5 patients in the control group.

^k^Data missing for 13 patients in the control group.

^l^Data missing for 1 patient in the monomicrobial group, 1 patient in the polymicrobial group and 11 patients in the control group.

^m^Data missing for 3 patients in the monomicrobial group, 3 patients in the polymicrobial group and 53 patients in the control group.

^n^Data missing for 1 patient in the monomicrobial group, 3 patients in the polymicrobial group and 29 patients in the control group.

^o^Highest preoperative values observed, except for hemoglobin where lowest preoperative values were used.

^p^Data missing for 1 patient in the polymicrobial group and 8 patients in the control group.

^q^Data missing for 5 patients in the control group.

^r^Data missing for 2 patients in the monomicrobial group, 2 patients in the polymicrobial group, and 50 patients in the control group.

^s^Data missing for 1 patient in the polymicrobial group and 8 patients in the control group. 1 patient in the monomicrobial group, 2 patients in the polymicrobial.

group and 20 patients in the control group had known CKD.

^t^Data missing for 1 patient in the polymicrobial group and 9 patients in the control group.

^u^Data missing for 3 patients in the monomicrobial group, 3 patients in the polymicrobial group, and 46 patients in the control group.

| **Supplementary table 3. Treatment and Outcome** | |  |  |
| --- | --- | --- | --- |
|  | **Monomicrobial (N = 10)^a^** | **Polymicrobial (N = 7)^b^** | **Controls (N = 185)^c^** |
| *Antibiotic treatment* |  |  |  |
| Anaerobic coverage^d^ | 10 (100) | 7 (100) | 184 (99.5) |
| Clindamycin before inclusion^e^ | 7 (70) | 6 (86) | 131 (71) |
| Betalactam and Clindamycin | 9 (90) | 6 (86) | 173 (94) |
| *Other treatment interventions* |  |  |  |
| Mechanical ventilation^d^ | 9 (90) | 7 (100) | 173 (94) |
| Renal replacement therapy^d,f^ | 2 (20) | 2 (29) | 24 (13) |
| Vasopressor/inotrope^d^ | 7 (70) | 5 (71) | 154 (83) |
| Hyperbaric oxygen^g^ | 7 (70) | 7 (100) | 161 (87) |
| Number of surgeries^d^ | 3 (1-14) | 3 (2-4) | 4 (1-13) |
| *Outcome* |  |  |  |
| Amputation^h^ | 2 (22) | 2 (29) | 15 (8) |
| 30-day mortality^i^ | 2 (20) | 4 (57) | 22 (12) |
| 90-day mortality^i^ | 4 (40) | 4 (57) | 34 (18) |

Data are presented as median (total range) or number (%).

^a^Clostridial NSTI.

^b^Polymicrobial NSTI with clostridia.

^c^Selected group of non-clostridial polymicrobial NSTIs.

^d^During first 7 days of stay at intensive care unit or high-dependency unit.

^e^Clindamycin given before admission to intensive care unit/high-dependency unit of the study hospital

^f^Preexisting chronic kidney disease among patients who received RRT: 1 patient in the polymicrobial group and 7 patients in the control group.

^g^At any time during hospitalization.

^h^Amputation of limb at primary hospital or during first 7 days of stay at intensive care unit or high-dependency unit at study hospital. Data missing for 1 patient in the monomicrobial group.

^i^Data missing for 1 patient in the control group.
